# Author Correction: A novel RGB-trichrome staining method for routine histological analysis of musculoskeletal tissues

**DOI:** 10.1038/s41598-021-98449-z

**Published:** 2021-09-15

**Authors:** Francisco Gaytan, Concepción Morales, Carlos Reymundo, Manuel Tena-Sempere

**Affiliations:** 1 Instituto Maimónides de Investigación Biomédica de Cordoba (IMIBIC), Cordoba, Spain; 2 Department of Cell Biology, Physiology and Immunology, Faculty of Medicine, University of Cordoba, Avda. Menéndez Pidal s/n. 14004, Cordoba, Spain; 3 Hospital Universitario Reina Sofia, Cordoba, Spain; 4 CIBER Fisiopatología de la Obesidad y Nutrición, Instituto de Salud Carlos III, 14004 Cordoba, Spain; 5 Department of Pathology, University of Cordoba, Cordoba, Spain

Correction to: *Scientific Reports* 10.1038/s41598-020-74031-x, published online 07 October 2020

The original version of this Article contained errors in the concentration of the components of the staining protocol.

As a result, in the Materials and Methods section, under subheading ‘Staining Protocol and Properties’,

"Thereafter, the sections were stained with 1% fast green FCF in distilled water for 20 min and rinsed in tap water for 5 min. Finally, the sections were stained with 1% sirius red in saturated aqueous solution of picric acid (i.e., picrosirius red) for 30 min, rinsed in two changes of 5 min in acidified water (1% acetic acid in tap water), dehydrated in two changes of 100% ethanol, cleared in xylene, and mounted in a resinous medium."

now reads:

“Thereafter, the sections were stained with 0.04% fast green FCF in distilled water for 20 min and rinsed in tap water for 5 min. Finally, the sections were stained with 0.1% sirius red in saturated aqueous solution of picric acid (i.e., picrosirius red) for 30 min, rinsed in two changes of 5 min in acidified water (1% acetic acid in tap water), dehydrated in two changes of 100% ethanol, cleared in xylene, and mounted in a resinous medium.”

The original Article has been corrected.

